# A Community-Based Study on Waist-to-Height Ratio: An Indicator for Systolic Hypertension in a Rural Community of Hilly Region

**DOI:** 10.7759/cureus.16014

**Published:** 2021-06-29

**Authors:** Santosh Kumar, Ravi Kant, Poonam Yadav, Kavitha Natarajan, Yogesh Bahurupi, Ashutosh Mishra

**Affiliations:** 1 Community and Family Medicine, All India Institute of Medical Sciences, Rishikesh, Rishikesh, IND; 2 General Medicine, All India Institute of Medical Sciences, Rishikesh, Rishikesh, IND; 3 College of Nursing, All India Institute of Medical Sciences, Rishikesh, Rishikesh, IND; 4 Physiology, Jawaharlal Institute of Postgraduate Medical Education and Research, Puducherry, IND

**Keywords:** body mass index, diastolic blood pressure, obesity, systolic blood pressure, waist to height ratio

## Abstract

The prevalence of hypertension gradually becomes a serious public health threat as it is a very pertinent risk factor for cardiovascular and cerebrovascular diseases. This study aims to estimate the prevalence of isolated systolic hypertension (ISH) among the hilly region's rural community and identify the indicators of ISH among study participants. A community-based cross-sectional study was conducted among 1220 participants in the rural community of the hilly region. A multistage random sampling technique was applied to recruit the participants. Demographic and anthropometric dimensions were measured to analyze the outcome of the study. The participants' mean age was 44.42 ± 15.54 years, with a majority of 822 female participants (67.40%). The prevalence of ISH was found as 27.45%. A statistically significant positive correlation (p < 0.05) of the waist-to-height ratio (WHtR) and body mass index (BMI) were observed with systolic blood pressure (SBP) in male as well as female participants, whereas BMI and WHtR had no correlation with diastolic blood pressure (DBP). Among female participants, the area under the curve (AUC) for BMI and WHtR was 0.604 (95% confidence interval 0.565-0.643, p-value = 0.020*) and 0.622 (95% confidence interval 0.584-0.660, p-value = 0.020*), respectively. Among male participants, the AUC for BMI and WHtR was 0.574 and 0.592, respectively. Hence, it cannot be considered very satisfactory. The increasing prevalence of ISH in a rural community is also a public health concern. At the preliminary stage, anthropocentric measurements are the primary tool for a family physician while treating the patients. This study concluded that WHtR is a better indicator than BMI for systolic hypertension. Although we have not observed a strong correlation of WHtR with systolic hypertension, it is required to perform future research to support this study's evidence.

## Introduction

Disease burden and associated risk factors have shifted significantly from communicable to non-communicable diseases (NCD) universally in the past two decades. Chronic NCDs are assuming increased importance among adults in both developing and developed countries. The increasing prevalence of hypertension gradually becomes a serious public health threat among NCDs as it is a very pertinent risk factor for cardiovascular and cerebrovascular diseases [1]. Isolated systolic hypertension (ISH) happens due to vascular tree elasticity, resulting in aggravation in SBP and normal DBP elasticity of vessels relevant in younger age groups [2]. Arterial stiffness/elasticity and stroke volume play a role in increasing the systolic blood pressure (SPB), pulse pressure amplification, and stroke volume, which was reported moderately higher in patients with ISH [2].

Many environmental and genetic factors are responsible for hypertension, such as gender, age, body mass index (BMI), diet, stress, physical activity, tobacco, and alcohol consumption. Among these factors, obesity, metabolic syndrome, and hereditary factors are important factors predisposing to hypertension. Many people go undiagnosed of hypertension because it rarely causes symptoms at the earlier stages, and people who are diagnosed may not have access to treatment, resulting in their inability to control their hypertension over a long time successfully. Approximately 17 million deaths contribute to cardiovascular disease in a year globally, which was nearly one-third of total deaths, and 9.4 million deaths account for only complications of hypertension worldwide every year. If hypertension were addressed and treated adequately at the earlier stage, there would be a significant social and economic impact [3].

A variety of anthropometric parameters such as BMI, waist circumference (WC), waist-to-height ratio (WHtR), and waist-hip ratio (WHR) are used for screening abdominal fat or total fat to assess risk for hypertension [4]. Central fat distribution has higher risks than those with peripheral fat in the risk population [5].

Hypertension is an iceberg disease, and scarcity of disease sometimes is misunderstood as the non-existence of the disease. After understanding modifiable risk factors' role, anthropometric parameters are the key to developing a clear and effective screening strategy in the community [6]. Assessment of risk of hypertension requires identification of a straightforward tool that can be used by a health worker in the screening program in the community. This study aims to assess the prevalence of ISH and its predictors in the study population of rural community settings of hilly regions.

## Materials and methods

Study setting

A cross-sectional study was conducted in Uttarakhand, also known as the “Indian central Himalayan region.” Uttarakhand is known for the high glacier of the Himalayas, with Nepal to the east, Himachal Pradesh near the West and northwest, and the Tibet region of China toward the north. Rishikesh is one of the six tehsils in the Dehradun district of Uttarakhand known as “Gateway to the Garhwal Himalayas” and “Yoga Capital of the World.” It lays approximately 25 kms from Haridwar and 43 kms from Dehradun, having an area of 500.73 km^2^. As per the 2011 census, Rishikesh has a population of 2,60,343 and 54,517 households. There are seven towns and 87 villages in Rishikesh Tehsil.

Study period

Data were collected from May 2018 to May 2019.

Study design

A community-based cross-sectional study was conducted from rural Rishikesh, Uttarakhand.

Sample size

Considering the previous prevalence of ISH (10.5%) by the Indian Council of Medical Research-India Diabetes (ICMR-INDIAB) study with a 95% confidence level, the relative precision of 20% of prevalence minimum sample size was 900. Since multistage random sampling was used, a design effect of 1.2 was applied to reduce biases.

n= Z2 p (1-p)/L2

where:

n = Sample size

Z = 1.96

p = Prevalence (from previous studies)

L = Allowable error

The sample size was estimated to be 1080, but 1220 study participants were included in the study.

Inclusion criteria

Adults aged from 18 to 90 years were included.

Exclusion criteria

Those who were pregnant, mentally ill, bedridden, seriously ill, having cardiovascular morbidity, stroke, and on hypertensive treatment were excluded from this study, and the person not willing to give consent was also included in the exclusion criteria.

Sampling technique

In this study, a multistage random sampling technique was used to recruit the participants. Rishikesh Tehsil was selected randomly among six tehsils of Dehradun district (Dehradun, Vikasnagar, Rishikesh, Chakrata, Kalsi, Tyuni) for this study. After selecting Rishikesh as the study area list of Rishikesh villages, there were 87 villages in Rishikesh as per the 2011 census. Using two-stage cluster random sampling and probability proportional to size, we selected 10 villages or clusters of 87 villages of Rishikesh Tehsil to obtain the desired sample for this study. We ensured that all persons in the sampling area had a similar probability of getting selected irrespective of their cluster, and from each cluster, the same number of persons had to be sampled.

In the first stage of cluster sampling, a list of 87 villages with population size and the cumulative population was prepared. Subsequently, the sampling interval (SI) was calculated by dividing the total cumulative population, one lakh thirty-seven thousand nine hundred and forty-three (1,37,943), by the number of clusters desired in the sample, which was 10 in number. Therefore, the SI obtained was 13794.3. A random number was generated from the cumulative population range, which was 7381, with the generation of random number first cluster selected for the study by matching the random number to the cumulative population, which was either equal to or exceeded the random number value. Subsequent clusters or villages were selected by adding a random start number (RS) to the SI. (second cluster = RS +SI, third cluster = RS + 2*SI, fourth cluster = RS + 3*SI, and so on). Athhoorwala, Bhogpur, Harrawala, Rishikesh, Jauligrant, Jeevan Wala, Khandraiwala, Mazri Grant, Rani Pokhari grant, and Shyampur were 10 villages or clusters selected for this study.

In the second stage of cluster sampling, individuals from each cluster were recruited using a simple random sampling method (lottery method). Total individuals selected from each cluster were 122 in number, and finally, we included 1220 individuals for this study from Rishikesh Tehsil.

Data collection

Informed consent was obtained from participants, and a pre-tested questionnaire was used to collect the data. Confidentiality of information was ensured to participants. Anthropometric dimensions like weight (kilograms) in a digital weighing scale, height (centimeter) in stadiometer, WC, and hip circumference (HC) (centimeter) using standard World Health Organization (WHO) protocols were measured. To get the detailed anthropometric indices, height (close to 0.001 m) and bodyweight (close to 0.1 kg) were documented in participants without shoes and only with light indoor clothes. The WC was measured between the lowermost border of the ribs and the iliac crest. The buttocks' maximum measured circumference will be the HC, the persons standing with feet placed together. Blood pressure was measured as per standard WHO protocol by using a mercury-free sphygmomanometer. Before the blood pressure measurement, participants were asked to relax for five minutes and then asked to sit in a relaxed position, with back supported, uncrossed legs with the bared upper arm. The arm needs to be supported at heart level. The cuff bladder was encircled 80% or more of the participant’s arm circumference. The mercury-free column was deflated at 2-3 mm per second. The first and last audible sound was recorded as systolic and diastolic pressure, respectively. Both the participants and investigator did not talk during the measurement of blood pressure.

Blood pressure was recorded twice. The intervals between the two recordings were one minute, and the mean of the measurements was recorded. The first reading in a series is usually the highest. Additional readings were taken if the difference between the first two was found greater than 5 mmHg.

Operational definitions

ISH: When SBP is ≥140 mmHg and diastolic blood pressure (DBP) is <90 mmHg [7].

For Asian populations, recent classification of BMI (kg/m^2^) was used to define “overweight (23-24.99 kg/m^2^)” and “obesity (>25 kg/m^2^)” [8]. Waist-to-height ratio (WHtR) defined as “WHtR < 0.5 (no risk), WHtR ≥ 0.5 < 0.6 (increased risk), and (WHtR ≥ 0.6 (very high risk)” [9].

Data analysis

Collected data was shifted to Statistical Package for the Social Sciences (SPSS) version 23.0 (IBM Corp., Armonk, NY), which was used for data analysis. Continuous variables were described as mean and standard deviation in the table. For categorical variables, cross-tabulation and frequency distribution were done. A Chi-square test was applied to determine association. Pearson’s correlation was applied to find out the correlation of ISH with anthropometric measures. In the receiver operating characteristic (ROC) curve, AUC was drawn to find out the best indicator for diagnosing hypertension. P-value was considered significant as <0.05.

## Results

The study population's mean age was 44.42 ± 15.54 years (males: 43.51 ± 17.43 years and females: 44.85 ± 14.525 years), with the majority of female participants as 67.40% (822/1220). The mean BMI among male and female participants was 25.690 ± 4.79 and 26.78 ± 5.005, respectively. The majority of the study population, 59.90% (731), belongs to the lower class, followed by the lower middle class, 31.10% (380), and 7.70% belong to the upper class. We also found a significant association of age, gender, and socioeconomic status with ISH (p-value < 0.01**) (Table 1).

**Table 1:** Demographic characteristics of participants and their association with isolated systolic hypertension n = 1220

Variables	Frequency (%)	P-value
Age (Mean ± SD)	44.42 ± 15.54	0.001
<25 yrs	169 (13.8)	
25-50 yrs	603 (49.4)	
50-75 yrs	417 (34.2)	
>75 yrs	31 (2.6)	
Gender		0.003
Male	398 (32.60)	
Female	822 (67.40)	
Socioeconomic status		0.000
Lower class	731 (59.90)	
Upper lower class	14 (1.10)	
Lower middle class	380 (31.10)	
Upper middle class	1 (0.10)	
Upper class	94 (7.70)	
Education		0.064
Primary education	247 (20.2)	
Up to higher education	300 (24.6)	
Graduation and post-graduation	585 (48)	
Professional education	88 (7.2)	
Marital status		0.56
Married	536 (43.9)	
Unmarried	677 (55.5)	
Others	7 (0.6)	
Chi-square test, p-value significant < 0.05*, < 0.01**		

Among them, 122 (10%) were known as diabetic, 85 (7%) were known as hypertensive, and 68 (5.60%) were on previous medication for diabetes and hypertension. The prevalence of ISH in the present study is 27.45%. We found a significant association of WHtR (p-value < 0.01**) and BMI (p-value < 0.01**) with ISH (Table 2).

**Table 2:** Association of clinical variables of participants with isolated systolic hypertension n = 1220 BMI, Body mass index.

Variables	Frequency (%)	P-value
Isolated systolic hypertension		
Yes	335 (27.45)	
No	885 (72.55)	
BMI		0.003
23-24.99 kg/m^2^	536 (44)	
>25 kg/m^2^	684 (56)	
Waist-to-height ratio		0.000
<0.5	251 (20.6)	
0.5-0.6	791 (64.8)	
>0.6	178 (14.6)	
On medication		0.000
Yes	68 (5.4)	
No	1152 (94.6)	
Diabetes		0.000
Yes	122 (10.1)	
No	1098 (90.9)	
Chi-square test, p-value significant < 0.05*, < 0.01**		

Summary of continuous variables such as weight, height, WC, SBP, diastolic blood pressure (DBP), and pulse rate has been shown with mean and standard deviation in Table 3.

**Table 3:** Summary of continuous variables n = 1220 BMI, Body mass index.

	Male	Female
Variables	Mean ± SD	
Age	43.51 ± 17.436	44.85 ± 14.525
Weight	63.01 ± 9.422	62.08 ± 9.44
Height	157.51 ± 10.956	152.92 ± 7.953
Waist circumference	84.82 ± 7.309	83.51 ± 7.488
Systolic BP	129.51 ± 19.293	128.88 ± 19.523
Diastolic BP	78.08 ± 10.966	77.12 ± 10.206
Pulse rate	74.95 ± 6.826	75.79 ± 7.346
Waist-to-height ratio	0.5406 ± 0.05486	0.5475 ± 0.05536
BMI	25.6904 ± 4.7958	26.7892 ± 5.00568

The BMI among female participants was higher compared to male participants. The mean WHtR among the study population was 0.540 ± 0.054 for males and 0.547 ± 0.055 for females.

SBP was significantly positively correlated (p-value < 0.01**) with WHtR and BMI among male and female participants, whereas BMI and WHtR had statistically insignificant (p > 0.05) and poor correlation with DBP among both males and females found (Table 4).

**Table 4:** Correlation of clinical variables with BMI and waist-to-height ratio among male and female participants BMI, Body mass index.

Pearson’s Correlation for Males		
Variables	BMI (r-Value)	Waist Height Ratio (r-Value)
Weight	0.700**	0.095
Height	-0.616**	-0.552**
Waist circumference	0.059	0.758**
Systolic BP	0.368**	0.354**
Diastolic BP	0.054	0.012
Pulse rate	-0.067	-0.084
Pearson’s Correlation for Females		
Weight	0.852**	0.231**
Height	-0.594**	-0.469**
Waist circumference	0.143**	0.865**
Systolic BP	0.373**	0.342**
Diastolic BP	0.002	0.035
Pulse rate	-0.007	-0.003
P-value significant < 0.05*, < 0.01**		

**Figure 1:**
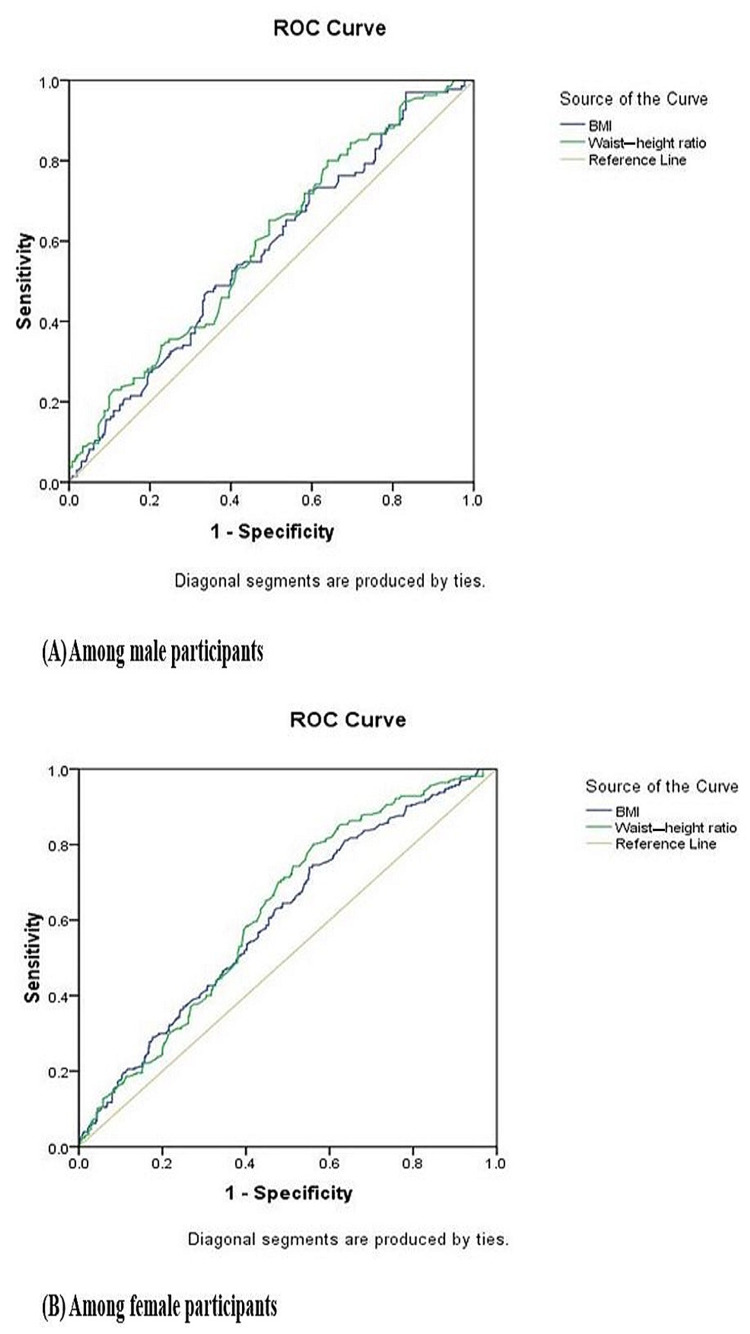
ROC curve for anthropometric parameter with systolic hypertension among participants ROC, Receiver operating characteristic curve.

Among female participants, the area under the curve (AUC) for BMI and WHtR was 0.604 (standard error: 26.3741, 95% confidence interval 0.565-0.643, p-value = 0.020*) and 0.622 (standard error: 0.5516, 95% confidence interval 0.584-0.660, p-value = 0.020*), respectively. Among male participants, the AUC for BMI and WHtR was 0.574 (standard error: 25.6445, 95% confidence interval 0.515-0.632, p-value = 0.030*) and 0.592 (standard error: 0.5467, 95% confidence interval 0.534-0.650, p-value = 0.030*), respectively. ROC curve indicated that WHtR can be the better indicator for diagnosing the hypertension than BMI among study participants (Figure 1).

## Discussion

The mortality and morbidity due to hypertension are rising among adults, especially in developing countries. This study was conducted among 1220 people residing in the rural hilly region of Uttarakhand. In the present study, the prevalence of ISH is 27.45%. A study conducted at the Lucknow district of North India in 2010 found that the prevalence of ISH among the young population was 4.5%, which is relatively lower than the present study [10]. Consistently, Malhotra et al. reported the prevalence of hypertension as 4.5% (JNC V [Joint National Committee on the Prevention, Detection, Evaluation, and Treatment of High Blood Pressure] criteria BP ≥ 140/90 mmHg) in a rural population of Haryana, North India, which has a similar geographical pattern to our study setting [11]. Kishore et al.’s study in India's capital region found that the prevalence of hypertension is 14.1%, higher in participants who are more than 35 years old than those who are less than 35 years old [12]. Nevertheless, it was relatively less than a study conducted in the north region, Chandigarh, where the prevalence of hypertension was 29.0%, consistent with the present study [13].

The total burden of type 2 diabetes mellitus (T2DM) in the present study was measured as 10%, comparable to various other studies as 8.03% and 7.5% of prevalence [14,15]. The variation in the prevalence could be explained by differences in age, geographical distribution, cultural practices, diet, sociodemographic pattern, alcohol, and tobacco consumption among the study population. In contrast to our findings, a survey conducted by Tripathy et al. reported the prevalence of isolated hypertension as 40.1%, higher than the present study [16].

The mean age of study participants in this study was 44.42 ± 15.54 years (males: 43.51 ± 17.43 years, females: 44.85 ± 14.52 years). It is comparable to the previous study conducted in Mysore, where the study subjects' mean age was 47.27 ± 10.31 years [17]. The mean SBP in the present study was 129.08 ± 19.44 mmHg (males: 129.51 ± 19.29 mmHg, females: 128.88 ± 19.52 mmHg). The mean DBP in the current study was 77.43 ± 10.4 mmHg (males: 78.08 ± 10.96 mmHg, females: 77.12 ± 10.20 mmHg) with no statistically significant difference between both the gender. This was comparable to the previous study conducted in Varanasi, India, where the mean SBP and DBP were 124.2 ± 15.0 and 83.4 ± 9.5, respectively [18]. Unlike our study, Singh et al. found a statistically significant difference for SBP and DBP between genders [18]. This variation may be described as urbanization, such as lifestyle changes and the participants' dietary patterns.

A previous study was conducted among the Punjab community residing in Delhi where the mean height among males and females was 166.2 ± 15.43 cm and 155.7 ± 5.78 cm, respectively, and the mean weight among the study population males and females was 67.8 ± 14.14 kgs and 63.2 ± 54.10 kgs, respectively, which were similar to the present study [19]. A study performed by Midha et al. found that the mean WC was 76.5 ± 11.3 (males: 78.9 ± 11.0 and females: 74.4 ± 11.1), which is consistent with our findings [20]. Huang et al. observed the mean pulse rate among study populations as 70.0 ± 9.0 beats per min, comparable to our study [21]. In the present study, females were found to have a higher BMI than males. Consistently, Dua et al. performed a study in the Capital Region of India and found that female participants had a higher BMI (27.7 ± 4.60) than male participants (25.8 ± 17.6) [19]. Concurrently, the mean WHtR (male: 0.547 ± 0.054 and female: 0.5475 ± 0.055) among study participants was similar to the study conducted by Verma et al. in Chandigarh, India. In that study, the WHtR (males: 0.054 ± 0.05; females: 0.55 ± 0.08) was indicated as a potent risk factor for hypertension [13].

In the present study, BMI and WHtR were positively correlated with systolic hypertension (SH) in male and female participants, whereas BMI and WHtR were not statistically significantly correlated with DBP in male or female participants. Consistently, Tesfaye et al. reported a positive correlation of SBP and DBP with BMI among population subgroups of Asia and Africa [22]. Kim et al. observed a positive correlation of SBP with WHtR, BMI, and body fat % among Korean adolescents [23]. As short stature is found to be the probable cause of the positive correlation between anthropometric measurements and SBP, height is known to be a strong indicator for the nutritional assessment at an early stage of life, and nutrition deficiency with short stature may predispose individuals to various metabolic changes in an organ system, cell structures, and physiology, which consequently leads to the obesity [24]. Anthropometric measurements were found to be associated with high screening parameters for an increase in blood pressure.

In contrast, Song et al. explained that BMI maintained a positive association with SBP and DBP [25]. In another study by Tawfik et al., where blood pressure was correlated with BMI, WHtR, and WC and SH, it was highly significant in patients with high WHtR but not significant with WC and BMI [26]. In calculating WHtR, height is an essential factor as short stature contributes to obesity and hypertension [27]. The mean height in the Indian population is 164.7 cm, whereas the mean height of the study participants is 154 cm in the present study. This difference may be the probable cause of the association of SH with WHtR. In ROC, an AUC for WHtR was 0.592 and for BMI was 0.514, which concluded that WHtR could be a better indicator for diagnosing hypertension than BMI among the study population in the present study, which could be substantiated with results of previous studies [28-30].

Adults who were found to have higher blood pressure values need to be followed up subsequently to treat and prevent associated complications. Factors such as salt intake, physical activity, tobacco, alcohol consumption, familial predisposition, and eating habits would be relevant for future surveys among hypertensive patients. Prevention of cardiovascular and cerebrovascular risk factors among hypertensive should be done as early as possible. Primordial prevention could be the only strategy to prevent NCDs in the community, mainly when resources are scarce. This study re-emphasizes the need to develop a preventive, promotive, and comprehensive plan to reduce hypertension by empowering the policymakers to realize the need for increased physical activity, healthy dietary habits, and lifestyle modification.

Strength of the study

It was a large-scale community-based study representing different age groups and socioeconomic strata in rural and hilly regions.

Prevalence of SH and their association between WHtR and BMI in the study population would reflect the increased prevalence of hypertension and related disorders as the obesity epidemic may further grow.

Limitation of the study

In the present study, environmental and occupational factors were not correlated with SH.

## Conclusions

The increasing prevalence of ISH among rural communities is also a public health challenge. Among obesity indicators, WHtR might be used as an initial screening tool for the risk of hypertension among adults due to its ease of measurement. A positive correlation between BMI and SH also indicates that lifestyle modification measures need to be adopted. Although a strong correlation of WHtR with SH was not noted hence, it is required to perform future research to support this study's evidence. Prevention and control of hypertension are complex processes. Therefore, multiple collaborations with the government, private sectors, and community should be made to reduce the risk of hypertension and its life-threatening complications.
